# Vaccines Hesitancy and Public Health

**DOI:** 10.3390/vaccines12101122

**Published:** 2024-09-30

**Authors:** Francisco Javier Pérez-Rivas, María Julia Ajejas Bazán

**Affiliations:** 1 Grupo de Investigación UCM “Salud Pública-Estilos de Vida, Metodología Enfermera y Cuidados en el Entorno Comunitario”, Departamento de Enfermería, Facultad de Enfermería, Fisioterapia y Podología, Universidad Complutense de Madrid, 28040 Madrid, Spain; majejas@ucm.es; 2 Red de Investigación en Cronicidad, Atención Primaria y Promoción de la Salud—RICAPPS—(RICORS), Instituto de Salud Carlos III, 28029 Madrid, Spain; 3 Instituto de Investigación Sanitaria Hospital 12 de Octubre (Imas12), 28041 Madrid, Spain; 4 Academia Central de la Defensa, Escuela Militar de Sanidad, Ministerio de Defensa, 28040 Madrid, Spain

We are delighted to present this editorial to close the Special Issue, ‘Vaccines Hesitancy and Public Health’, which we have had the honour of coordinating.

Although vaccination is widely recognised as one of the most effective public health interventions, in recent years several factors have created barriers to acceptance that transcend cultural and geographic boundaries [1]. This reluctance to vaccinate has increased since the COVID-19 pandemic situation unfolded, largely influenced by the media and the pressure exerted by anti-vaccine movements on social media [2].

This Special Issue brings together 16 articles that address this problem from multiple perspectives, providing an exhaustive and up-to-date analysis of the causes and implications of, as well as strategies for tackling, this problem. Several published articles have assessed the factors that have influenced vaccination against COVID-19, highlighting how mistrust in health systems, a short time to market, the availability of several vaccines with different levels of efficacy and safety, and misinformation spread by the media and social networks have contributed to the relatively low vaccination coverage rates achieved. This Issue also contributes to a better understanding of the factors influencing reluctance in different social groups (some of them in vulnerable situations, at certain life stages, or with specific health problems) and in different geographical areas (different vaccines in different countries). In addition, several articles highlight the influence of personal factors, such as psychological stress or mental health problems, on vaccine acceptance and the need for personalised approaches that take these factors into account. Another very relevant aspect addressed in some of the articles is the reluctance on the part of health professionals. In some cases, this is associated with significant educational gaps; this is especially worrying considering the influence that the beliefs and attitudes of professionals have on the decision of patients and their families to receive vaccination. Also covered in this Issue are aspects of innovation, such as the use of a text message reminder and recall system to improve the management of vaccination programmes. Finally, it should be noted that most studies propose a number of strategies to improve vaccine uptake, focusing mainly on improved communication and the adaptation of personalised approaches to the situation and context of each individual, involving health professionals and community leaders as sources of trust. In this regard, this Issue contains an article of interest on how mega-studies can provide valuable information on the most effective interventions in terms of reducing vaccine reluctance. 

As Professor Salleras has recently pointed out, reluctance is not a static behaviour, conduct or psychological state, but a dynamic process ranging from the total or partial acceptance of vaccines to the absolute or selective rejection of vaccines [3]. This process is influenced by numerous factors that generate hesitancy to vaccinate, which need to be addressed in a comprehensive and coordinated manner. A comprehensive approach involving all areas and contexts of society is essential to address what the WHO considered in 2010 to be one of the top 10 global public health problems [4].

Finally, we would like to thank and acknowledge all the authors of the manuscripts included in this Special Issue, as well as the members of the *Vaccines* editorial team for their administrative support. 
